# Gene expression networks and functionally enriched pathways involved in the response of domestic chicken to acute heat stress

**DOI:** 10.3389/fgene.2023.1102136

**Published:** 2023-05-02

**Authors:** Sevda Hosseinzadeh, Karim Hasanpur

**Affiliations:** Department of Animal Science, Faculty of Agriculture, University of Tabriz, Tabriz, Iran

**Keywords:** heat stress, WGCNA, machine learning, eGWAS, chicken

## Abstract

Heat stress in poultry houses, especially in warm areas, is one of the main environmental factors that restrict the growth of broilers or laying performance of layers, suppresses the immune system, and deteriorates egg quality and feed conversion ratio. The molecular mechanisms underlying the response of chicken to acute heat stress (AHS) have not been comprehensively elucidated. Therefore, the main object of the current work was to investigate the liver gene expression profile of chickens under AHS in comparison with their corresponding control groups, using four RNA-seq datasets. The meta-analysis, GO and KEGG pathway enrichment, WGCNA, machine-learning, and eGWAS analyses were performed. The results revealed 77 meta-genes that were mainly related to protein biosynthesis, protein folding, and protein transport between cellular organelles. In other words, under AHS, the expression of genes involving in the structure of rough reticulum membrane and in the process of protein folding was adversely influenced. In addition, genes related to biological processes such as “response to unfolded proteins,” “response to reticulum stress” and “ERAD pathway” were differentially regulated. We introduce here a couple of genes such as HSPA5, SSR1, SDF2L1, and SEC23B, as the most significantly differentiated under AHS, which could be used as bio-signatures of AHS. Besides the mentioned genes, the main findings of the current work may shed light to the identification of the effects of AHS on gene expression profiling of domestic chicken as well as the adaptive response of chicken to environmental stresses.

## Introduction

Heat stress is one of the main concerns of the poultry industry, especially in warm areas, as it causes major economic losses in both layer and broiler farms. Intensive genetic selection in breeding programs has led to an increased growth rate and metabolism. However, the development of the chicken thermoregulatory system cannot be matched with the growth rate, making it difficult for industrial chickens to regulate their body heat as temperatures fluctuate (Havenstein et al., 2003). In addition, chickens are sensitive to high temperatures due to the absence of sweat glands (Loyau et al., 2013), feather cover, and high density in commercial rearing facilities (Brugaletta et al., 2022). The most common negative effects of heat stress (HS) on chickens are on growth performance, egg production and quality (Barrett et al., 2019; Awad et al., 2020), feed intake, appetite hormone regulation (Mazzoni et al., 2022), oxidative properties (Altan et al., 2003), intestinal health, immune response (Deng et al., 2012), body temperature (Van Goor et al., 2015), and increased mortality (Khosravinia, 2016). It is estimated that the HS could lead to annual economic losses of $128 to $165 million in the United States poultry industry (St-Pierre et al., 2003). Selective breeding of HS-resistant chicken is a suitable scenario for producing a well adaptable strains (Radwan, 2020). Therefore, the characterization of genetic biomarkers associated with resistance to HS will pave the way for selective breeding to generate the HS-resistant chickens. Transcriptome comparison may elucidate the genetic base of HS (Sun et al., 2015). The liver has an important role in general metabolism, synthesis of bile and proteins, and maintenance of homeostasis (Rui, 2014), and is more vulnerable to HS than other organs as HS triggers oxidative stress (Lin et al., 2006). The liver is also impacted by the increased production of biochemical anti-oxidants (Mahmoud and Edens, 2003). Previous studies have shown that some genes in liver tissue undergo significant expression modifications under HS as compared to the normal condition. For example, HSP70, HSP90 (Radwan, 2020), HSP90B1, HSPA5 (Sánchez et al., 2022), MX1, TLX1, HSPB9 (Wang et al., 2020), HSP70, HSPA5 (Wang et al., 2021), ANGPTL4 (Lan et al., 2016) have been introduced as biomarkers for HS in chickens. These genes have not been identified from a sole, comprehensive research but from multiple similar studies, each of which had identified only one or, at most, a couple of the mentioned genes. In other words, there is little consensus on the results of the studies that aim to address the same scientific question. Therefore, there should be a statistical methodology to merge the findings of multiple independent but similar studies. Meta-analysis is a quantitative and systematic method for combining the *p*-values obtained from the analysis of RNA-seq data from multiple related studies. Meta-analysis could overcome the issues that arise from the low number of biological replicates in the experiments, and could result to an improved statistical power due to the larger sample size that come from multiple datasets (Tseng et al., 2012; Rau et al., 2014). By meta-analyzing, the results of multiple small but related studies could be combined to attain a pooled estimate that is closest to the common truth. By relieving the sources of disagreement among the related results, meta-analysis of multiple studies makes interesting relationships come to light. The Fisher approach, which is usually implemented in the meta-analyses, has been proven to be an appropriate method for the combining the *p*-values, and is useful for the identification of differentially expressed genes (DEGs) and novel biomarkers (Calduch-Giner et al., 2014; Landry and Sirard, 2018; Lindholm-Perry et al., 2020). Because of the complex nature of the biological systems in which many genes or biological agents interact with each other, there is an increased request for researches that aim to elucidate the complex interaction of genes that have been identified as biologically important. The study of gene co-expression networks helps to categorize genes with the same expression pattern. The interaction of genes could more easily be predicted by analyzing the modules than by analyzing the genes themselves (Cho et al., 2012). Therefore, weighted gene co-expression network analysis (WGCNA) could be used as a desirable approach for discovering the co-expressed genes and nodes (Ramayo-Caldas et al., 2018; Wang et al., 2020; Sánchez et al., 2022). In the present study, RNA-seq data from four different but related datasets were assessed to identify the DEGs, meta-genes, modules, and hub-genes in the chicken liver tissue under acute heat stress (AHS). Finally, we introduced the major genes associated with AHS.

## Materials and methods

### RNA-seq data collection from databases

The Sequence Read Archive (SRA) repository of the National Center for Biotechnology Information (https://www.ncbi.nlm.nih.gov/sra) was screened precisely to find the appropriate RNA-seq datasets that address the research question of the current work using the keywords “Gallus gallus,” “chicken,” “liver” and “acute heat stress”. We could find only four RNA-seq datasets in which the AHS was the main treatment. Detailed information of the four selected datasets can be found in Table 1. In addition, the accession numbers of the used samples are reported in In Supplementary Table S1 **.**

**TABLE 1:** Information of the used datasets for the analyses.

Dataset accession number	Group	Number of runs	Raw reads	Alignment rate (%)	References	Breed	Sex	Age at sample collection (week)	Duration of heat stress	Sequence length (PE/SE)
SRP268422-A	Heat stress	4	35611146 -47290916	88.47-91.57	Wang et al. (2020)	Leghorn	not collected	2	4 h	51-100 (PE)
	Control	4	37009786-52258461	89.45-90.94						
SRP268422-B	Heat stress	4	40234382-44711995	88.63-90.03	Wang et al. (2020)	Fayoumi	not collected	2	4 h	54-100 (PE)
	Control	4	22254064-43734081	90.94-91.18						
ERP014602-A	Heat stress	4	27335951-33532021	92.86-94.88	Lan et al. (2016)	Broiler	male	3	3 h	35-100 (SE)
	Control	4	20135467-30196233	92.44-95.49						
ERP014602-B	Heat stress	4	26448275-31900734	91.43-93.87	Lan et al. (2016)	Fayoumi	male	3	3 h	42-100 (SE)
	Control	4	26358318-35802997	93.18-94.27						

### Analyses of individual datasets

After retrieving the RNA-seq datasets, fastq-dump tool of SRA-Toolkit version 2.9.6 (Staff, 2011) was employed to convert the SRA files into the FASTQ format. The data quality was assessed using the fastQC tool version 0.12.1 (Andrews, 2010) (available at https://www.bioinformatics.babraham.ac.uk/projects/fastqc/). The low quality reads were eliminated using the Trimmomatic (version 0.39) software (Bolger et al., 2014) (available at http://www.usadellab.org/cms/?page=trimmomatic) with ILLUMINACLIP, SLIDING WINDOW (3-5: 20-28), CROP (3-10), AVGQUAL (20-25) and MINLEN (40-45) options. The implemented values varied among the datasets according to their quality metrics. The trimmed reads were mapped onto the reference genome Gallus_gallus.GRCg6a (https://asia.ensembl.org/Gallus_gallus/info/index) using HISAT2 (version 2.2.1) software (Kim et al., 2019) (available at https://daehwankimlab.github.io/hisat2). The expression count matrix was generated using HTSeq-count (version 0.9.1) (Anders et al., 2015). Then, the DESeq2 package (version 3.16) (Love et al., 2014) was used to identify the DEGs using the default parameters. To characterize the gene symbols, biotypes, and positions of each transcript, we uploaded the DESeq2 results and the gtf file (http://ftp.ensembl.org/pub/release-104/gtf/gallus_gallus/Gallus_gallus.GRCg6a.104.gtf.gz) to galaxy (available at https://usegalaxy.eu/) and we used the Annotate DESeq2/DEXSeq output tables (version 1.1.0) for annotation.

### Meta-analysis

The metaRNASeq package version 1.0.2 (Marot and Rau, 2013) in *R* was used to identify the meta-genes. First, raw *p*-values and log2-fold change values of all expressed genes of all four datasets were gathered in a new file. Then, *p*-values were combined via the fishcomb function. Comparing the sign (positive or negative) of the log2-fold change of the genes across the four datasets revealed the consistency or inconsistency of the expression of genes in all datasets. Finally, genes with consistent expression in all datasets, with adjusted *p*-value ≤0.05 in at least one dataset, and meta-analysis *p*-value ≤0.05 were considered as meta-genes.

### Gene ontology, KEGG pathway analysis, and protein-protein interaction

Meta-genes were interpreted based on Gene Ontology (GO) and Kyoto Encyclopedia of Genes and Genomes (KEGG) Pathways by DAVID web base software (https://david.ncifcrf.gov). Terms with *p*-value ≤0.05, FDR ≤0.2, and a fold enrichment >2 were considered as significant. Furthermore, protein-protein interaction (PPI) network was created using the STRING database (https://string-db.org/) to construct the network and identify the hub-genes. Cytoscape software (version 3.7.2) (Shannon et al., 2003) was used to visualize the retrieved networks.

### Weighted gene co-expression network analysis

The R package Weighted Gene Co-expression Network Analysis (WGCNA) (version 1.71) (Langfelder and Horvath, 2008) was used to identify the co-expressed networks, modules, and hub-genes. To alleviate the influence of noise when calculating the correlations based on the read counts, we filtered out the genes with read counts less than 10 in more than 90% of the samples, in order to reduce the sampling differences. The variance-stabilizing transformations (VSTs) output from Deseq2 were also used as input. However, in the case of using VST data, the variations caused by batch effects or other covariates could not be accounted for. Therefore, the function “removeBatchEffect” was used to eliminate the batch variations by package limma (version 3.50.3). In the first step, excessive missing values and outlier samples were examined with the options “goodSamplesGenes” and “hclust”. Soft threshold power of 0.9 was selected based on the scale-free topology index (*R* ^2^) (Zhang and Horvath, 2005) and, thus, the “pickSoftThreshold” option was used to calculate the adjacency matrix. The Pearson correlation coefficients between each pair of genes were calculated. The adjacency matrix was converted to a Topological Overlap Matrix (TOM) to minimize the effects of noise. Then, the corresponding dissimilarity matrix (1-TOM) was generated. A dynamic tree cut (DTC) algorithm was used to detect and construct the gene co-expression modules with the following parameters; cut height of 0.975, minimum module size of 30 genes, DeepSplit of 2, and hybrid method.

To identify the hub-genes, the module eigengenes were calculated using the “moduleEigengenes” function, and the “intramodularConnectivity” process was used to calculate both the intramodular connectivity (k_within_) and the total connectivity (k_total_). Then, the “corPvalueStudent” process was used for the identification of hub-genes based on the *p*-values (Degli Esposti et al., 2019). In addition, the “chooseTopHubInEachModule” and “chooseOneHubInEachModule” options were used to identify the hub-genes within each module.

### Functional analysis of meta-genes and co-expressed modules

After identifying the meta-genes and significant modules, the common genes between them were identified using venn diagram (http://bioinformatics.psb.ugent.be/webtools/Venn/). The common significant meta-genes were interpreted by enrichment analysis methods based on the KEGG pathway and GO using the ClueGO plugin of Cytoscape software (version 3.7.2) (Bindea et al., 2009), and subsequently, only pathways with Bonferroni Step Down corrected *p*-values <0.01 were considered as significantly enriched.

### Machine-learning

The identified meta-genes with their corresponding expression values were submitted to Machine-Learning for the selection of the most important HS-related genes. Additionally, the chicken breed, age at sample collection, and duration of the HS were used as three attributes. This was accomplished based on seven weighting algorithms, including Uncertainty, Chi-Squared, Relief, Gini index, Rule, Information Gain, and Gain Ratio. Meta-genes with average weighting values above 0.7 across all seven algorithms were analyzed via Rapid Miner software (version 9.9) for 10-fold cross-validation using stratified sampling with Decision Tree (Accuracy, Gain Ratio, Gini Index, and Information Gain criterion), Random Forest (Accuracy and Gain Ratio criterion), Deep Learning (Tanh and Rectifier criterion), and Naive Bayes.

### SNP calling and eGWAS analyses

**FIGURE 1:**
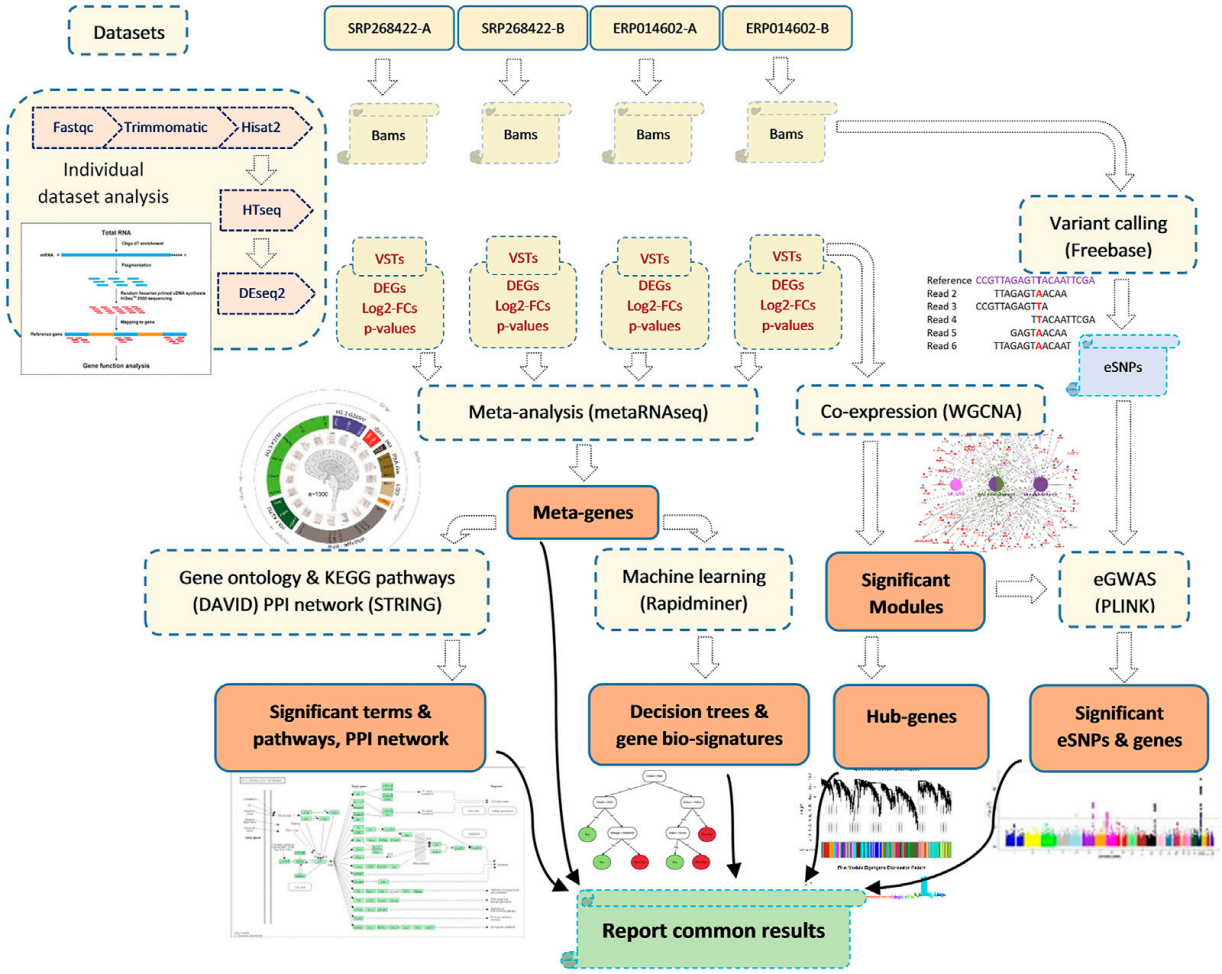
Overview of the steps gone for the data collection, preprocessing, analyzing and results combination. The collected datasets belonged to four similar RNA-seq experiments that the main object of all of them was to address the effect of acute heat stress on chicken liver gene expression profile. The results that obtained commonly from all individual dataset analysis, meta-analysis, weighted gene-co-expression network analysis (WGCNA), machine learning and expression-based genome wide association analysis (eGWAS) were the ultimate base of the identification of key genes related to the response of chicken to acute heat stress.

For SNP calling, the Galaxy platform (available at https://usegalaxy.eu/) was used with Genotype-variants outline (available at https://github.com/cfarkas/Genotype-variants). After sorting the BAM files with samtools sort (galaxy version 2.0.4) (https://samtools.github.io/hts-specs/), we used FreeBayes (galaxy version 1.3.6+ galaxy0) (https://github.com/ekg/freebayes) for variant calling. We then used VCFfilter (galaxy version 1.0.0_rc3+galaxy3) (https://github.com/ekg/vcflib) to keep only the SNPs with a depth of more than 25 reads and a quality of more than 30. The transcript IDs harboring each variant were retrieved using VCFannotate (galaxy version 1.0.0_rc1+galaxy0) (GitHub. https://github.com/ekg/vcflib). For expression based genome wide association study (eGWAS), we used 22,197 eSNPs located on all analyzed transcripts. The expression values (read counts) of the transcripts were required to be above 10 in at least 10% of the samples to be included in the eGWAS. The quality assessment of the SNP genotypes was carried out by PLINK software (version 1.9) (Purcell et al., 2007). The nine significant modules that were output by WGCNA software were considered as nine traits, and the eigenvalues of the 32 samples for each of the significant modules were considered as dependent variables (phenotypes). A linear model was employed in the “assoc” analysis of PLINK. The significant eSNPs were visualized using an R package called CMplot (version 4.2.0) (Yin et al., 2021). Figure 1 illustrates the flowchart of the analysis steps of the four RNA-seq datasets in the present study. It should be mentioned that, all gene feature were reported in the current manuscript and supplementary files were according to the Gallus_gallus.GRCg6a genome assembly of domestic chicken.

### Validation of the results

Two additional RNA-seq datasets related to chicken livers under chronic heat stress (CHS) were used to validate the identified 77 meta-genes. The mentioned datasets were downloaded from the NCBI website with accession numbers ERP014602 and SRP100368 with 8 and 16 samples, respectively. Half of the samples in each of the mentioned datasets were from the chickens treated with 35 degrees of Celsius for 7-8 h and for 7 days, whereas the next halves were considered as control groups. The accession numbers of the used runs for the validation analysis can be found in Supplementary Table S2. The analysis pipeline was as same as mentioned above for the analysis of the AHS datasets.

## Results

### Individual dataset analysis

We examined four selected RNA-seq datasets and identified 41, 115, 39, and 12 DEGs for SRP268422-A, SRP268422-B, ERP014602-A, and ERP014602-B datasets, respectively. Of which, 24, 83, 10, and 5 were downregulated while 17, 32, 29, and 7 were upregulated DEGs, respectively. In Supplementary File S1, detailed information about the results of the four RNA-seq datasets analyses are provided. The primary aim of analyzing four similar, related datasets was to discover the DEGs that were commonly present in all of the studied datasets. Therefore, we were most interested in the common DEGs across the results of four individual datasets. We, found, however, a little number of common DEGs. In Supplementary Figure S1, the Venn diagram of the DEGs identified in the four individual experiments has been shown.

### Meta-analysis

As mentioned above, the results obtained from the four RNA-seq datasets did not support each other. Therefore, the implementation of meta-analysis seemed necessary. As such, we combined the results of the four abovementioned datasets and carried out a meta-analysis. Finally, a total of 77 significant meta-genes were identified. Ten out of the 77 meta-genes did not have gene symbols, while the remaining 67 meta-genes possessed known gene symbols. Supplementary File S2 includes the detailed results of the significant meta-genes from the meta-analysis of four RNA-seq datasets.

### Functional enrichment analysis

**FIGURE 2:**
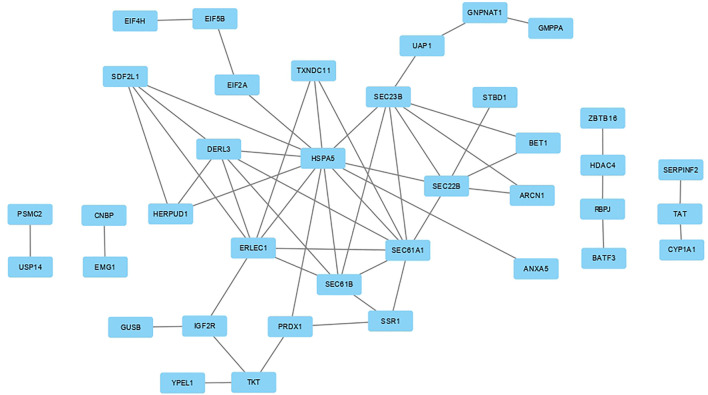
Protein-protein interaction (PPI) network analysis based on meta-genes. By definition, the meta-genes were those gene that were significantly differentially expressed between the chickens under acute heat stress and their corresponding control group as well as those that were consistently expressed across the four datasets (with the same direction of expression) and were significant in the meta-analysis. The PPI network was constructed using the STRING website.

We used the DAVID database to perform KEGG pathway and GO analyses on meta-genes to characterize their molecular function (MF), biological process (BP), and cellular component (CC). The results revealed 3, 1, and 3 significant GO terms for BPs, CCs, and MFs, respectively. The terms “posttranslational protein targeting to membrane translocation,” “endoplasmic reticulum unfolded protein response” and “ubiquitin-dependent ERAD pathway” were three significant BP terms. The “endoplasmic reticulum membrane” CC and there MF terms “identical protein binding,” “ribosome binding,” and “misfolded protein binding” were significantly enriched by meta-genes. Through this process, four significant KEGG pathways including “Protein processing in endoplasmic reticulum,” “Protein export,” “Biosynthesis of nucleotide sugars,” and “Amino sugar and nucleotide sugar metabolism” were revealed to associate with AHS. A thorough information on the KEGG pathways and GO terms have been provided in Table 2. The PPI network of the meta-genes was also studied and the resulted network was visualized using the Cytoscape. In Figure 2 the visualized PPI network of the meta-genes is shown.

**TABLE 2:** Gene ontology and KEGG pathway enrichment results of meta-genes in the comparison of chickens under acute heat stress and their corresponding control groups.

Category	Term	Count	%	*p*-value	Genes	List total	Pop Hits	Pop total	Fold enrichment	FDR
Biological Process	GO:0031204∼posttranslational protein targeting to membrane, translocation	3	4.5	3.1E-04	SEC61A1, HSPA5, SEC61B	53	7	13,312	107.6	0.0589
	GO:0030968∼endoplasmic reticulum unfolded protein response	4	6.1	3.4E-04	SERP1, HSPA5, DERL3, HERPUD1	53	35	13,312	28.7	0.0589
	GO:0030433∼ubiquitin-dependent ERAD pathway	4	6.1	0.0013	HSPA5, DERL3, SEC61B, HERPUD1	53	55	13,312	18.3	0.1491
Cellular component	GO:0005789∼endoplasmic reticulum membrane	10	15.2	1.1E-05	SEC61A1, SERP1, SDF2L1, DERL3, CYP1A1, SSR1, SELENOK, SEC22B, SEC23B, HERPUD1	55	385	14,587	6.9	9.5E-04
Molecular function	GO:0042802∼identical protein binding	12	18.2	2.9E-04	HDAC4, EMG1, SALL1, PRDX1, TAT, GNPNAT1, UAP1, SELENOK, NMRAL1, IGF2R, PTPRG, JMJD6	53	811	13,048	3.6	0.0388
	GO:0043022∼ribosome binding	4	6.1	0.0011	SEC61A1, HSPA5, SEC61B, EIF2A	53	51	13,048	19.3	0.0733
	GO:0051787∼misfolded protein binding	3	4.5	0.0026	SDF2L1, HSPA5, DERL3	53	19	13,048	38.9	0.1148
KEGG pathway	gga04141:Protein processing in endoplasmic reticulum	8	12.1	8.0E-06	SEC61A1, ERLEC1, HSPA5, DERL3, SSR1, SEC61B, SEC23B, HERPUD1	27	151	5011	9.8	2.8E-04
	gga03060:Protein export	3	4.5	0.0067	SEC61A1, HSPA5, SEC61B	27	24	5011	23.2	0.1166
	gga01250:Biosynthesis of nucleotide sugars	3	4.5	0.0154	GMPPA, GNPNAT1, UAP1	27	37	5011	15.0	0.1799
	gga00520:Amino sugar and nucleotide sugar metabolism	3	4.5	0.0252	GMPPA, GNPNAT1, UAP1	27	48	5011	11.6	0.2207

### Weighted gene co-expression network analysis and identification of the hub-genes

**FIGURE 3:**
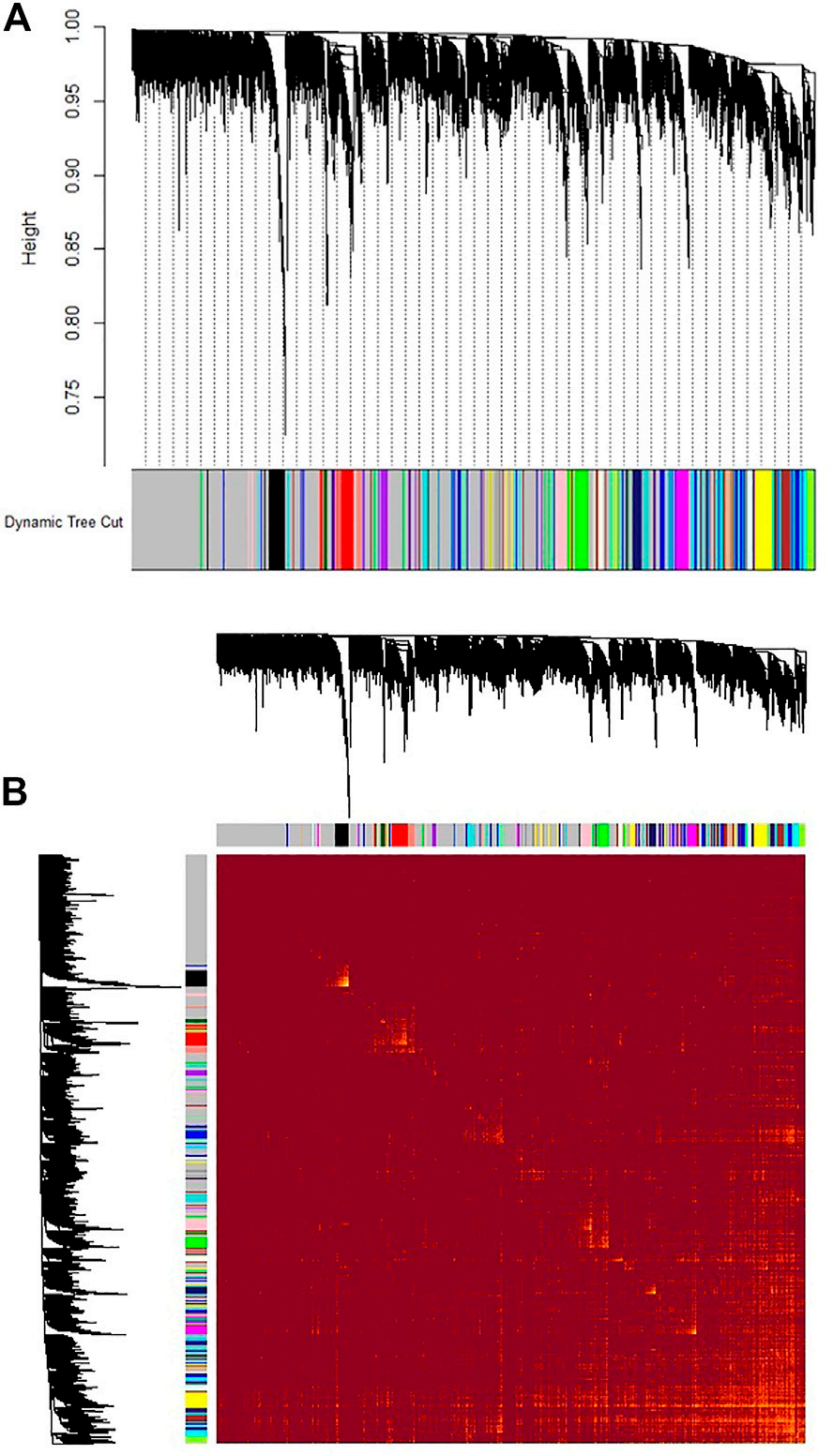
Gene co-expression modules which grouped the reliably expressed genes (with read count > 10 in more than 10% of the samples) into 22 modules. **(A)** The height (*y*-axis) indicates the co-expression distance and the *x*-axis corresponds to genes. Colors represent the 22 modules. The gray module represent genes that assigned to neither of the 22 modules. **(B)** Heatmap plot of topological overlap in the gene network. Each row and column corresponds to a gene, light color denotes low topological overlap, and progressively darker red denotes higher topological overlap. Darker squares along the diagonal correspond to modules. The gene dendrogram and module assignment are shown along the left and top.

**FIGURE 4:**
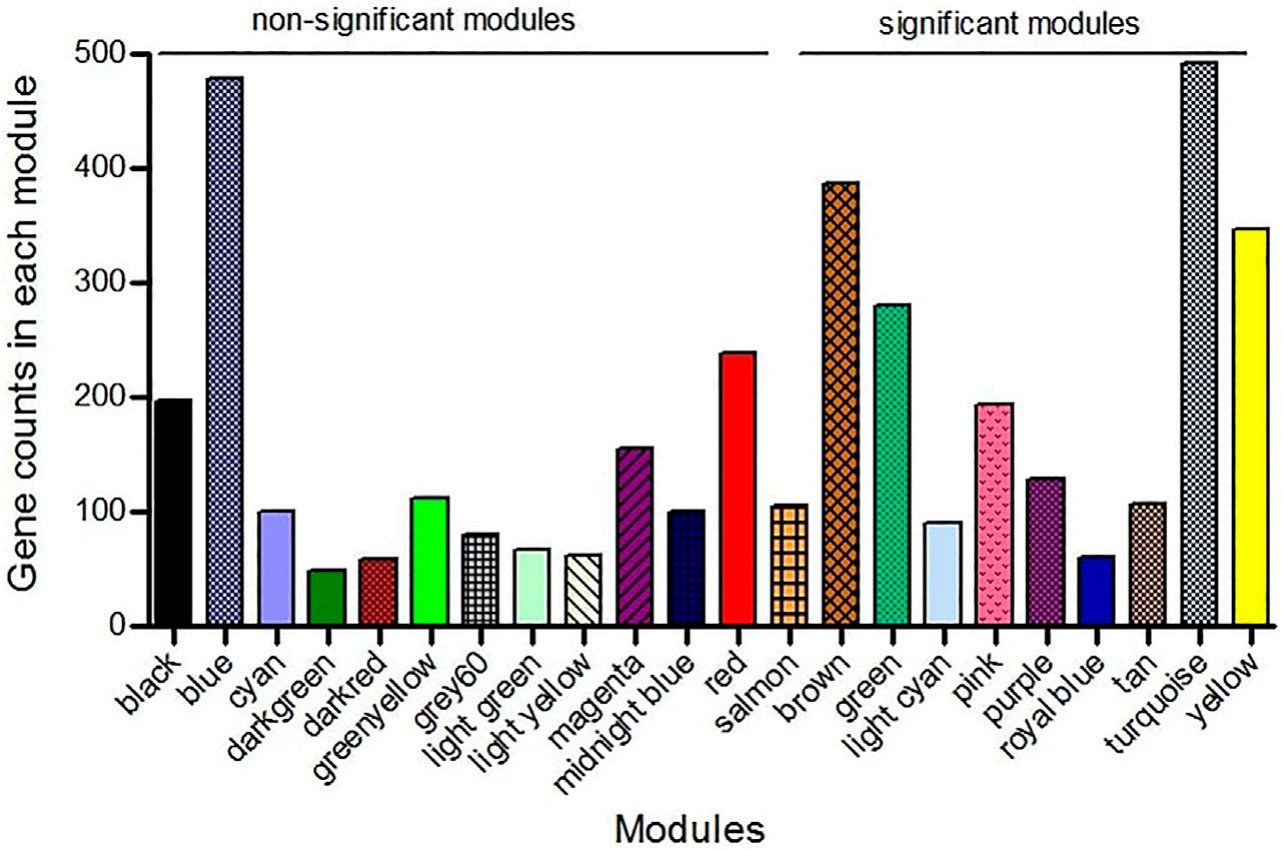
The result of weighted gene co-expression network analysis (WGCNA) that detected 22 modules in which the co-expressed genes were grouped in the same modules. The nine significant modules (*p*-value <0.05) were specified from the non-significant modules.

**FIGURE 5:**
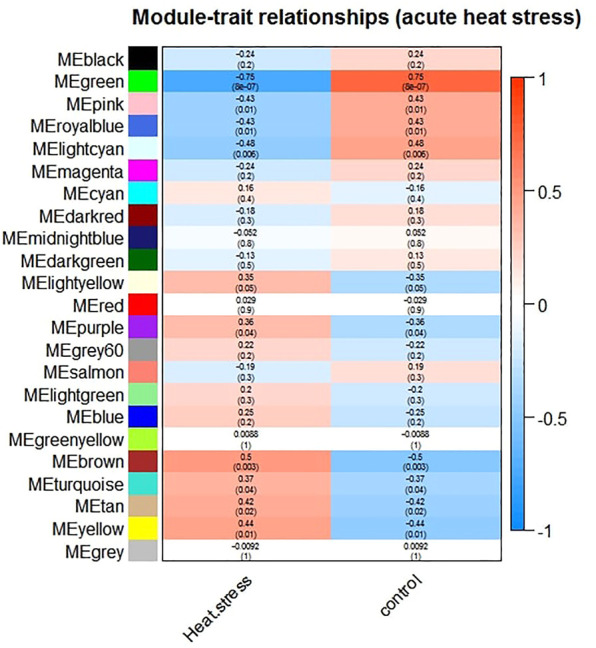
Module-trait relationships between the identified modules and treatment groups (acute heat treated versus non-treated control groups). The correlation between the traits and the module eigengenes was used for the calculation of the relationship. The red color indicate strong positive correlation while the blue color indicate strong negative correlation. Rows represent module eigengene and columns represent the treatment groups. The first row within each cell represent the correlation and the second one represent the significance level (*p*-value).

The expression values of 7829 genes were used as input data for WGCNA (Supplementary File S3). In the first step, excessive missing values and outlier samples were examined, and outlier samples were removed **(** Supplementary Figure S2A **)**, and a value of 6 was determined as a soft threshold power (Supplementary Figure S2B). We used the dynamic tree cut algorithm to construct the modules. Input genes were grouped into 22 modules, ranging in size from 49 to 492 genes, along with 3940 genes that could not be grouped in any of the modules and therefore were called as unassigned (Figures 3–A). The hierarchical clustering of genes using the topological overlap matrix (TOM) is shown in Figures 3–B. Nine out of the 22 modules including green (*r* = −0.75, *p*-value = 8e-7), pink (*r* = −0.43, *p*-value = 0.01), royal blue (*r* = −0.43, *p*-value = 0.01), light cyan (*r* = −0.48, *p*-value = 0.006), purple (*r* = +0.36, *p*-value = 0.04), brown (*r* = +0.5, *p*-value = 0.003), turquoise (*r* = +0.37, *p*-value = 0.04), tan (*r* = +0.42, *p*-value = 0.02), and yellow (*r* = +0.44, *p*-value = 0.01) modules were identified as particularly significant. In Figure 4 and (Supplementary File S4) the genes counts and names within each of the 22 modules, and in Figure 5 the Pearson correlation coefficient and *p*-values of the identified modules are reported. Thereafter, hub-genes were identified per module based on the *p*-values using the “corPvalueStudent” (Supplementary File S5). In Table 3 the identified five hub-genes per module are reported.

**TABLE 3:** Top five hub genes, Top hub, and One hub genes in significant modules for acute heat stress.

Significant modules	Five top hub genes (*p*-value)	One hub gene	Top hub gene
Brown	KDR (2.9E-14)	KDR	KDR
	ITPRIP (1.3E-12)		
	DGKD (1.3E-12)		
	FLT4 (5.0E-12)		
	CCDC50 (7.9E-12)		
Green	HSPA5 (1.3E-15)	HSPA5	BET1
	SSR1 (3.8E-15)		
	BET1 (5.3E-13)		
	SDF2L1 (1.3E-12)		
	HYOU1 (3.8E-12)		
light cyan	ITPR2 (5.9E-13)	ITPR2	ITPR2
	AREL1 (2.7E-11)		
	SNRNP27 (3.6E-11)		
	PPIB (9.9E-11)		
	TRIP12 (1.6E-10)		
Pink	LRRC34 (1.8E-16)	LRRC34	METTL23
	METTL23 (1.0E-15)		
	CEP68 (2.1E-15)		
	MRPS7 (2.6E-15)		
	METTL5 (5.9E-15)		
Purple	OGT (1.7E-14)	OGT	CCNL2
	SRSF11 (7.1E-14)		
	CCNL2 (1.3E-13)		
	RBM3 (3.9E-12)		
	AP2M1 (2.2E-11)		
royal blue	NDUFB1 (1.2E-14)	NDUFB1	NDUFB1
	DBI (1.2E-13)		
	ATP5MC1 (1.4E-12)		
	IQGAP1 (4.3E-12)		
	UQCRHL (8.6E-12)		
tan	KIF1B (1.4E-13)	KIF1B	KIF1B
	FEM1A (1.7E-12)		
	TMEM94 (2.9E-12)		
	ANGEL1 (3.2E-11)		
	DOCK4 (1.2E-10)		
turquoise	PDGFRB (2.1E-14)	PDGFRB	GFRA1
	DUSP16 (3.9E-14)		
	MYH11 (1.9E-12)		
	LIFR (8.6E-12)		
	DNAJC12 (1.27E-11)		
yellow	CDC42BPB (1.9E-14)	CDC42BPB	VPS13C
	CPT1A (7.8E-14)		
	VPS13C (1.3E-12)		
	PIKFYVE (1.4E-12)		
	ABCA10 (1.5E-11)		

### Functional effects of the meta-genes and co-expressed modules

**FIGURE 6:**
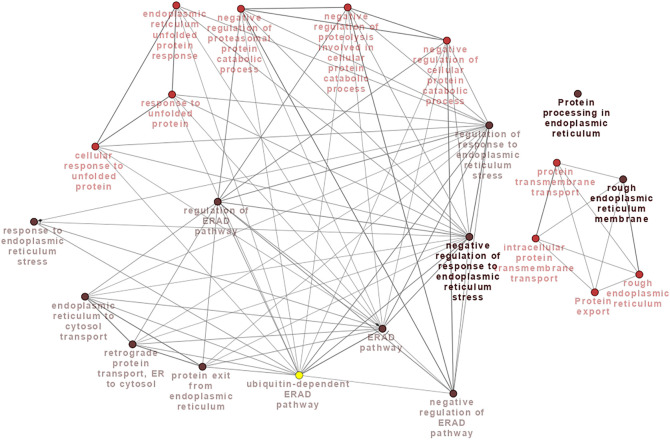
The association of the identified significant terms which enriched by the meta-genes.

Meta-analysis relieved the sample size limitation, while the WGCNA identified genes with high expression correlation. For functional enrichment analysis, we assigned significant common genes between the modules and meta-genes (Supplementary File S6 **)** using the ClueGO plugin of Cytoscape software. As a result of pathway enrichment analysis, “Protein processing in endoplasmic reticulum,” and “Protein export” were found to be significant KEEG pathways, and “rough endoplasmic reticulum” and “rough endoplasmic reticulum membrane” were two significant CC terms. “Protein transmembrane transport,” “intracellular protein transmembrane transport,” “response to endoplasmic reticulum stress,” “response to unfolded protein,” “endoplasmic reticulum to cytosol transport,” “endoplasmic reticulum unfolded protein response,” “negative regulation of response to endoplasmic reticulum stress,” “regulation of response to endoplasmic reticulum stress,” “cellular response to unfolded protein,” “negative regulation of cellular protein catabolic process,” “protein exit from endoplasmic reticulum,” “ERAD pathway,” “retrograde protein transport, ER to cytosol,” “negative regulation of proteolysis involved in cellular protein catabolic process,” “regulation of ERAD pathway,” “negative regulation of ERAD pathway,” “negative regulation of proteasomal protein catabolic process” and “ubiquitin-dependent ERAD pathway” were significant terms for BP category. The connections among the terms are illustrated in Figure 6. In addition, the detailed information about the significantly enriched terms are reported in Supplementary File S7.

### Machine learning and validation of the identified meta-genes

**FIGURE 7:**
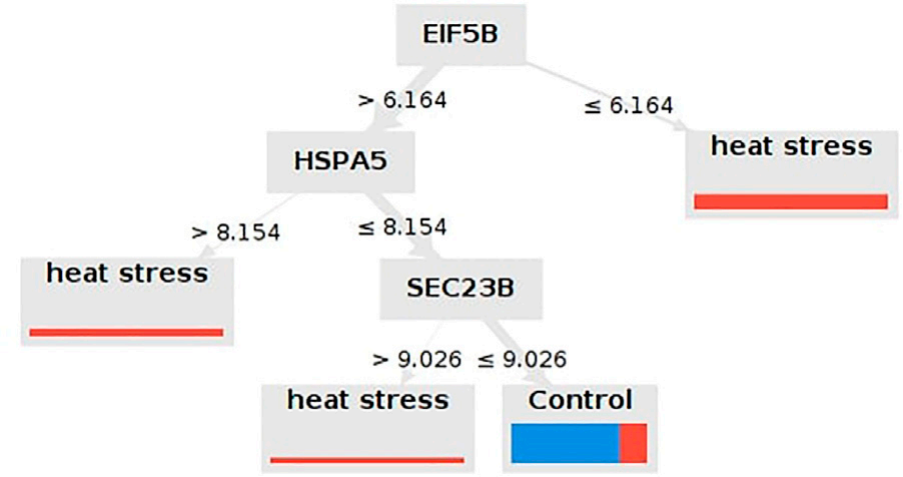
Decision Tree that identified the most important biomarkers for the differentiation of chickens under cute heat stress from those under normal rearing condition. Only the Decision Tree of the most accurate criterion (i.e., Gain Ratio) was shown here.

Seventeen meta-genes showed an average weighting values greater than 0.7. For breed, age at sampling, and duration of HS, however, the average weighting values were less than 0.7. In Supplementary Table S3, the weights of the 17 meta-genes obtained from the 7 different machine learning algorithms are reported. Ten models were applied to the dataset. The performances of the models are reported in Table 4. According to the cross validation results, the Decision Tree with the Gain Ratio criterion, the Random Forest with the Accuracy criterion, and Deep Learning with the Maxout parameter showed acceptably high accuracy. The Decision Tree identified key meta-genes that classify the chickens under AHS or control conditions based on the expression values. Here, three genes including EIF5B, HSPA5, and SEC23B are introduced as biomarkers for AHS as they were included in all Decision Trees with the Gain Ratio, Accuracy, Gini Index, and Information Gain criteria. The accuracy range of the mentioned models were 67.50%+/-37.26%, 57.50%+/-40.64%, 65.00%+/-43.23%, and 55.00%+/-42.61%, respectively. Thus, based on the Gain Ratio, if the expression value of EIF5B was greater than 6.164, the samples would fell into the AHS, otherwise, the expression value of HSPA5 must be taken into account (Figure 7; Supplementary Figure S3). The important role of these three genes on AHS was also identified earlier in the WGCNA analysis, as all of them included in the significant green module as hub-genes. The rediscovery of the mentioned three genes by the Decision Tree models confirms the important association of them with AHS.

**TABLE 4:** The performance of machine learning models in acute heat stress in chickens with ten-fold cross validation.

Model	Accuracy	Sensitivity	Specificity	F_measure (%)	Precision	AUC
Random Forest with Accuracy criterion	60.00%+/-47.57%	56.25%	68.75%	60.00	64.29%	0.600±0.384
Random Forest with Gain Ratio criterion	60.00%+/-41.68%	50.00%	75.00%	57.14	66.67%	0.500±0.397
Decision Tree with Gain Ratio criterion	67.50%+/-37.26%	62.50%	75.00%	66.67	71.43%	0.725±0.302
Decision Tree with Accuracy criterion	57.50%+/-40.64%	50.00%	56.25%	51.61	53.33%	0.625±0.358
Decision Tree with Gini Index criterion	65.00%+/-43.23%	56.25%	75.00%	62.07	69.23%	0.700±0.299
Decision Tree with Information Gain criterion	55.00%+/-42.61%	50.00%	56.25%	51.61	53.33%	0.525±0.380
Deep Learning with Tanh parameter	27.50%+/-37.96%	56.25%	0.00%	43.90	36.00%	0.200±0.251
Deep Learning with Rectifier parameter	40.00%+/-34.79%	75.00%	12.50%	57.14	46.15%	0.450±0.394
Deep Learning with Maxout parameter	47.50%+/-41.28%	56.25%	31.25%	50.00	45.00%	0.400±0.384
Naive Bayes	21.90%+/-20.37%	15.00%+/-22.36%	31.67%+/-33.54%	13.79	15.00%+/-22.36%	0.239±0.237

### eGWAS analyses

**FIGURE 8:**
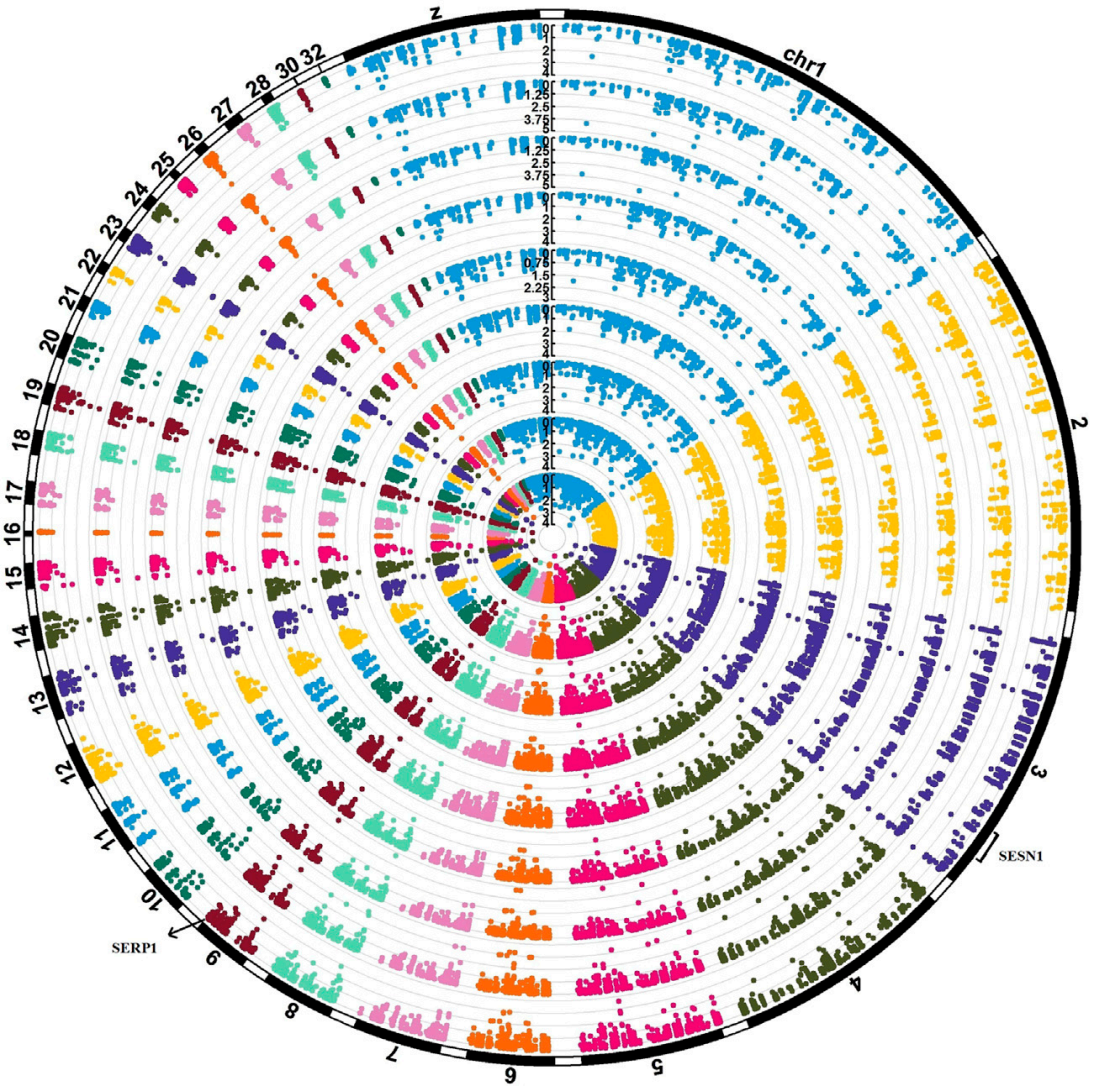
Circular Manhattan plot that visualized the association of eSNPs with the eigenvalues of nine modules (layers of circles). The eSNPs were called from the RNA-seq data of 32 chickens. Four eSNPs in SESN1 meta-gene (positions chr3:67138413, 67135835, 67137358, 67137810) and one eSNP on the SERP1 meta-gene (position chr9:23805255) were found to be associated with all nine modules.

To identify significant SNPs associated with nine significant modules, eGWAS was conducted. As a result, we identified 406 significant eSNPs (*p*-values ≤0.05). It is noteworthy to mention that most of the significant eSNPs were associated with two modules, including yellow and green. In Supplementary File S8 significant levels of all 406 eSNPs on all nine significant modules are provided. A total of 134 significant eSNPs located on the genes that included within the significant modules, and 52 of them were significantly associated with all nine significant modules. In Figure 8, a circular Manhattan plot illustrating the association of eSNPs with nine modules (layers of circles) is shown. We identified one eSNP on gene SERP1 (position chr9:23805255) and four eSNPs on gene SESN1 (positions chr3:67138413, 67135835, 67137358, 67137810). The SERP1 was a meta-genes which consistently downregulated in AHS, while SESN1 was a meta-genes which consistently upregulated in AHS as compared with the control group.

To summarize our results from the five employed approaches and make relevant conclusions, we decided to focus on genes that were commonly observed as meta-genes and included within the significant terms, pathways, and modules, especially those that were identified as hub-genes. Furthermore, the meta-genes with significant eSNPs that highlighted by Machine Learning were considered as more important. In Supplementary Figure S4 and Supplementary File S9, the resulting Venn diagram has been reported. Based on the summary of our results, we introduce important genes associated with AHS including; HSPA5, SSR1, SDF2L1, SEC23B, SERP1, and SESN1.

### Validation of the results

**FIGURE 9:**
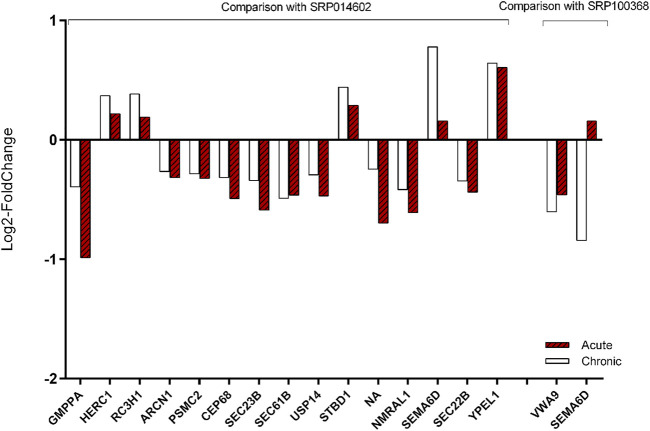
Validation of the expression modification of 17 meta-genes by two additional RNA-seq datasets. The expression fold changes of the 17 meta-genes were strongly in agreement with those in the two validation experiments. Since we could not find other datasets from the acute heat stress experiments, we used two datasets that belonged to chronic heat stress.

Fifteen and two meta-genes out of the 77 meta-genes were identified as differentially expressed (*p*-value <0.05) in the two additional datasets (i.e., ERP014602 and SRP100368, respectively). Surprisingly, only one of the 17 genes showed no match with the results of the current work. In other words, the log2-fold change of the 16 meta-genes in all four AHS datasets were as in the same direction as in the validation datasets (CHS). In Supplementary File S10 the log2-fold change of the 77 meta-genes in two validation datasets are provided. In Figure 9, the expression concordance of the 17 identified meta-genes between the AHS datasets and CHS datasets were shown. We also compared the expression pattern of the six most associated meta-genes we introduced above (i.e., HSPA5, SSR1, SDF2L1, SEC23B, SERP1, and SESN1). The expression pattern of five of them were matched between the AHS and CHS datasets.

## Discussion

High temperatures negatively impact the production efficiency of commercial chicken via injurious physiological (Mujahid et al., 2007; Sandner et al., 2020), biochemical (Xie et al., 2015), and immune capacity (Park et al., 2019). Unlike the commercial chicken breeds, AHS does not adversely affect the local chicken breeds (Lawrence and Wall, 2014; Porto-Neto et al., 2014). Because, in commercial chicken breeds the thermoregulatory system is not compatible with the metabolism rate. Additionally, the commercial strains do not have the genetic potential to develop heat tolerance, and are not able to regulate their body temperature during ambient temperature fluctuations (Havenstein et al., 2003; Coble et al., 2014; Lan et al., 2016; Fleming et al., 2017). We studied the RNA-seq data that originated from both broilers and layers (Leghorn and Fayoumi), which all of them were commercially important, in order to conduct a comprehensive study that encompassed both major chicken types and three different breeds. As such, the obtained results might be generalized for other commercial breeds.

Selective breeding with the aim of generating HS-tolerant chicken might be the best choice to produce a well adaptable strains (Mack et al., 2013; Misztal, 2017; Radwan and Mahrous, 2019; Radwan, 2020). Accordingly, the identifying key genes and variants related to AHS is essential in genetic selection. Therefore, we investigated four RNA-seq datasets, all of which aimed to elucidate the effect of AHS on chicken whole transcriptase gene expression profile, in order to gain deep insights into the key regulatory and hub-genes associated with AHS. To this end, we carried out multiple approaches including meta-analysis, GO and KEGG pathway enrichment analysis, WGCNA, machine learning, and eGWAS. The obtained results from the individual datasets analyses did not support each other, and there were only few common DEGs across the four experiments (Supplementary Figure S1). Therefore, the identification of meta-genes through meta-analysis was critically necessary as it is an efficient statistical method that diminishes the limitations that arise from the small sample size within each of the individual datasets and, by increasing the statistical power, identifies the key genes with small effects (Farhadian et al., 2018; Keel et al., 2018; Benny et al., 2019; Jiang et al., 2022; Yuan et al., 2022). Both WGCNA and eGWAS are also efficient approaches for identifying hub-genes and significantly associated alleles. As such, we found 77 meta-genes in the current work. These meta-genes were included within the significant modules, and showed commonness with the hub-genes that were identified by WGCNA, and jointly were observed as important genes within the Decision Trees and eGWAS (Supplementary Figure S4). The overall consequence of the used approaches led to the identifying a couple of significant meta-genes including HSPA5, SSR1, SDF2L1, SEC23B, SERP1, and SESN1 that could be considered as bio-signatures of AHS. The mentioned meta-genes were within the green module (the module with the highest negative correlation with AHS; *r* = −0.75, *p*-value = 8e-7) and were confirmed by GO analysis, as well. HSPA5 is an endoplasmic reticulum chaperone complexes and located in the “endoplasmic reticulum lumen” which plays important roles in the KEGG pathways “protein processing in endoplasmic reticulum,” and “protein export”. Based on the GO analysis, it can be postulated that all significant terms related with the functions of meta-genes were associated with the protein folding and complementation of the final structure of the proteins. In other words, the results indicate that in the presence of AHS the three-dimension structure of the proteins are adversely affected, or misfolded. In accordance with the present study, the results of the previous studies show that the HSPA5 gene is suppressed under AHS in the liver of commercial chicken breeds (Schmidt et al., 2015; Wang et al., 2020; 2021). On the contrary, HSPA5 did not show differential expression under HS in the native chickens (Sánchez et al., 2022). HSPA5 has been shown to be differentially expressed in other tissues, for example, upregulated in the testis (Wang et al., 2014; 2015) and small yellow follicles (Cheng et al., 2018) and downregulated in the spleen under HS (Zhang et al., 2019). It has been reported that a decrease in the transcription of HSPA5 in response to endoplasmic reticulum stress leads to a decrease in translation, protein folding, protein assembly, and protein transport through the cell (Lee, 2005; Wang et al., 2019) and suppresses the immunity (Zhang et al., 2019) and homeostasis (Kim et al., 2021) in chickens. The suppression of the expression of SSR1 under AHS was observed in the current study. SSR1 locate on the “endoplasmic reticulum membrane” and involve in “protein processing in endoplasmic reticulum”. The protein encoded by the SSR1 gene acts as a glycosylated membrane of the endoplasmic reticulum and involve in the translocation of proteins across the endoplasmic reticulum membrane. In a similar way, the SDF2L1 gene also plays an important role in the structure of the endoplasmic reticulum membrane, and acts as a binding agent on the misfolded protein. The expression of the SDF2L1 gene was suppressed under the AHS via “unfolded protein response”. Previous studies have shown that SDF2L1 interacts with folding enzymes and endoplasmic reticulum chaperones (Meunier et al., 2002; Bies et al., 2004; Tongaonkar and Selsted, 2009). It interacts with HSPA5 to regulate the activity of chaperones (Wang et al., 2017; Hanafusa et al., 2019; Conner et al., 2020). Furthermore, the SEC23B meta-gene, which was downregulated by AHS and resides within the green module, was found to be a bio-signature of AHS in the Decision Tree. It locates on the “endoplasmic reticulum membrane” and plays a significant role in the “protein processing in endoplasmic reticulum” pathway. SEC23B involves in protein secretion (Saito et al., 2009) by activating the formation of transport vesicles from the endoplasmic reticulum (Fromme et al., 2008) and, therefore, can activate the genes related to the innate immune system (Fox et al., 2010). Along with the above mentioned meta-genes, the expression of other important meta-genes (e.g., EIF5B, USP14, GMPPA, SEC61A1, HDAC4, PTPRG, IGF2R, SEC61B, DERL3, PRDX1, EMG1, ERLEC1, NMRAL1, SELENOK, HERPUD1, JMJD6, TAT, EIF2A, PEX10, and UAP1) which also involve in the process of protein synthesis, were modified significantly under the AHS and need to be further investigated. For example, EIF5B catalyzes the joining of the ribosome 40S and 60S subunits and plays an important role in translation initiation (Lee et al., 1999). USP14 is an associated subunit of the proteasome and is a physiological inhibitor of the ERAD pathway and plays a critical role in the innate immune defense (Puente et al., 2003). SEC61 has cooperated with protein SEC62, SEC63, and HSPA5 to enable post-translational transport of small proteins (Haßdenteufel et al., 2018) and has a role in cellular calcium homeostasis (Schubert et al., 2018). HDAC4 is Responsible for histone deacetylation for epigenetic repression and therefore have role in cell cycle progression, transcriptional regulation, and developmental events (Wang et al., 2022). SEC61 channel mediates transport of polypeptides across the endoplasmic reticulum (Meacock et al., 2002) and involve in the biogenesis of proteins (McGilvray et al., 2020). DERL3 retrotranslocate the misfolded glycoproteins into the cytosol (Lilley and Ploegh, 2004).

We identified one significant eSNP on the SERP1 and four significant eSNPs on the SESN1. The SESN1 gene was one of the meta-genes of the brown module, which has a positive correlation with AHS (*r* = +0.5, *p*-value = 0.003). SESN1 has been associated with HS in cardiac and skeletal muscle and has a role in MAP Kinase signaling pathway (Srikanth et al., 2019). SESN1 also acts in hyperthermia resistance and antioxidant firewall (Budanov et al., 2004). SESN1 involve in the regulation of metabolism, energy homeostasis, cell growth, and viability under various cellular stresses (Velasco-Miguel et al., 1999; Budanov et al., 2002). SERP1 is induced under the presence of stressors and interacts with the molecular chaperone calnexin, which can control early membrane protein biogenesis (Faria et al., 2012). SERP1 is one of the unfolded protein response genes (Vickers et al., 2017). It seems that the overexpression of SERP1 can alleviate the acute injury of liver (Cai et al., 2022). SERP1 plays a significant role in “endoplasmic reticulum unfolded protein response,” and “endoplasmic reticulum membrane” based on BP and CC, respectively. It seems that the identified eSNPs cause allele-specific gene expression and are associated with the AHS. Overall, our results support the previous findings on AHS in chickens. For example, ANGPTL4 was also identified in the study by Coble et al. (2014), which was one of the meta-genes within the yellow modules of the current study which harbor three significant eSNPs. HSPA5, HSPA8, TTC7A, CMPK2, TTC7A, HSPH1, CEMIP, ADAMTS15, TMEM255A, FAMM222A, and JMJD6 genes were identified as important related genes in the study by Kim et al. (2021). HSPA5, TTC7A, HSPH1, HSPA8, TTC7A, and FAMM222A were within the genes of the green module, and CEMIP, ADAMTS15, TMEM255A, MBOAT2 were within the genes of brown, turquoise, and light cyan modules, respectively. CMPK2 and JMJD6 were two meta-genes that the second was a member of the green module and involved in the MF term “ribosome binding”. Both HSPA5 and ANKRD9 were identified as related key genes in the study by Wang et al. (2021). ANKRD9, which harbored three significant eSNPs, was a member of the turquoise module in the current study. Barreto Sánchez et al. (2022) identified CPT1A and ANGPTL4 as potential candidate genes related with AHS, and we identified both of them as either hub-gene (CPT1A) or a member of the yellow module (ANGPTL4). Although we discovered the introduced genes via multiple bioinformatics approaches and the accuracy of the identification of them seems great, we strongly suggest further detailed wet-lab experiments to elucidate the effects of AHS on the introduced genes. Moreover, further research into the regulatory effects of the identified key genes is also recommended.

## Conclusion

In the present study, DEGs, meta-genes, genes co-expression networks, hub-genes, and alleles associated with AHS were identified. We found that under AHS, some components of the endoplasmic reticulum chaperone complexes (HSPA5, SDF2L1) were suppressed, which may result to the disruption of non-covalent folding and unfolding of the proteins. The translocation of proteins across the endoplasmic reticulum membrane and protein secretion also seem to be disrupted by the downregulation of SSR1 and SEC23B genes. In general, we postulate that the AHS leads to the disruption of protein structure, protein binding, protein translocation, protein formation, and degradation of the misfolded proteins.

## Data Availability

The datasets presented in this study can be found in online repositories. The names of the repository/repositories and accession number(s) can be found in the article/Supplementary Material.
