# Supplementary figures and images for: Gene expression networks and functionally enriched pathways involved in the response of domestic chicken to acute heat stress
Source: Front Genet. 2023 May 2;14:1102136. doi: 10.3389/fgene.2023.1102136 (PMC10185895; doi:10.3389/fgene.2023.1102136)

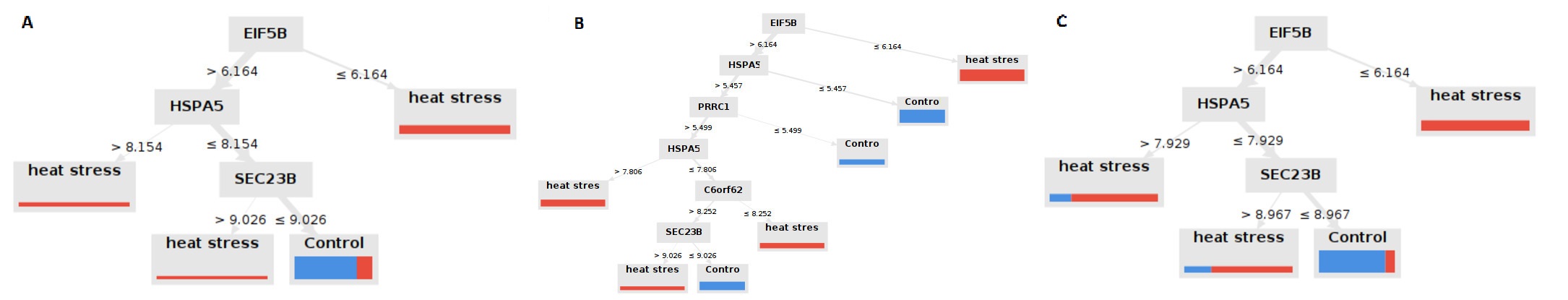

Supplement: Supplementary file 1 [file Image3.JPEG]

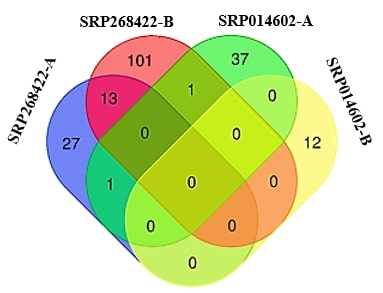

Supplement: Supplementary file 6 [file Image1.JPEG]

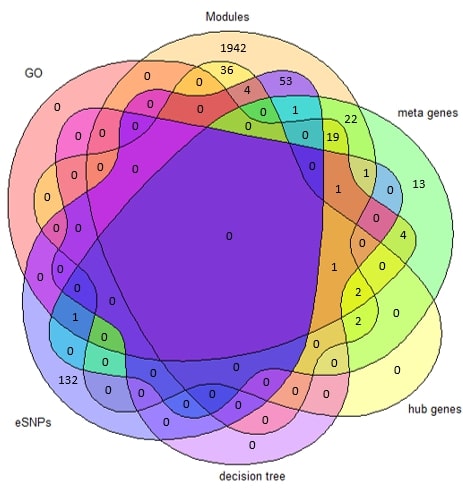

Supplement: Supplementary file 7 [file Image4.JPEG]

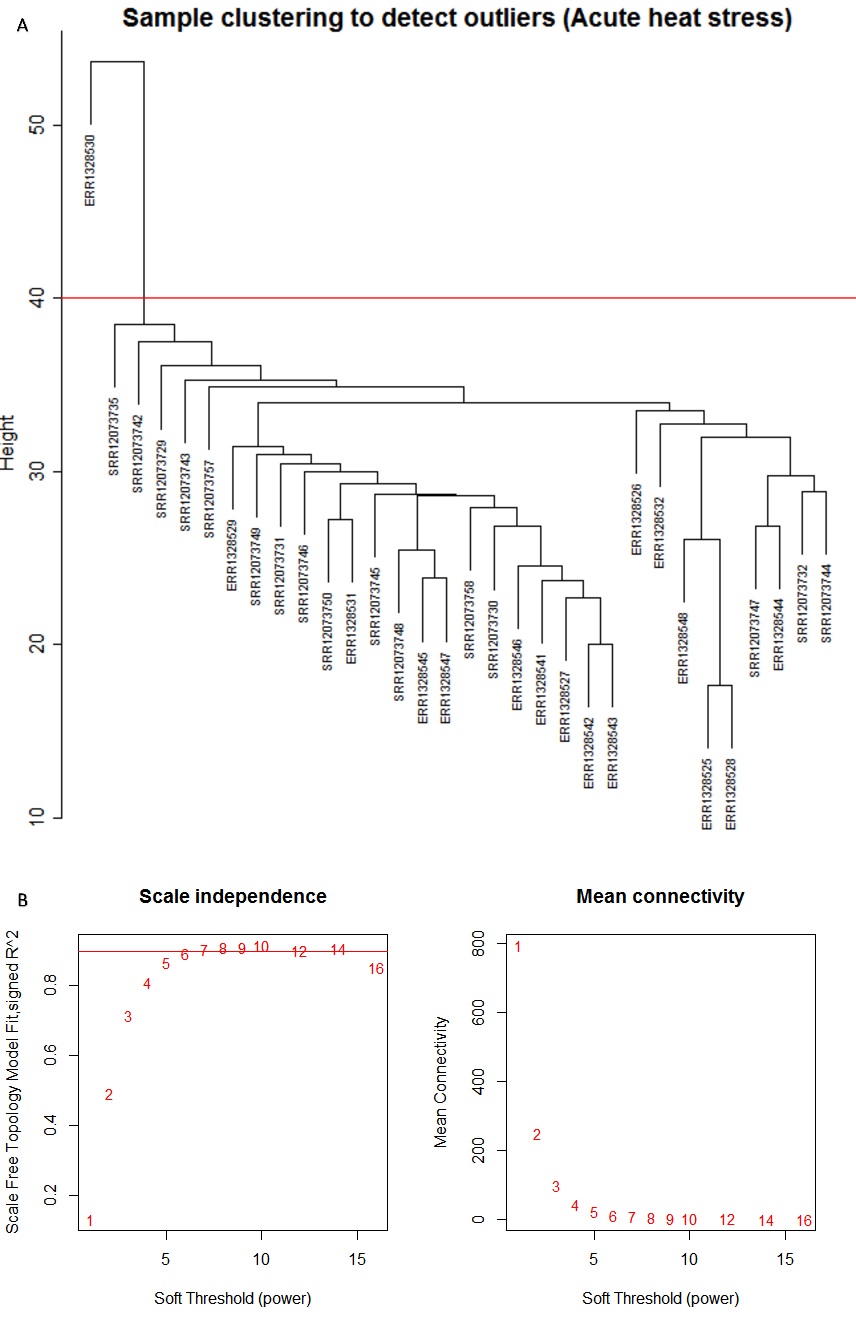

Supplement: Supplementary file 9 [file Image2.JPEG]
