# Supplementary material for: Gene expression networks and functionally enriched pathways involved in the response of domestic chicken to acute heat stress
Source: Front Genet. 2023 May 2;14:1102136. doi: 10.3389/fgene.2023.1102136 (PMC10185895; doi:10.3389/fgene.2023.1102136)
Supplement: Supplementary file 3 [file Table1.docx]

| Supplementary Table 1. The accession numbers of the used samples for the main analysis | | | | | |
| --- | --- | --- | --- | --- | --- |
| Dataset accession number | Group | Accession number of runs | | | |
| [SRP268422-A](https://trace.ncbi.nlm.nih.gov/Traces/sra?study=SRP268422) | Heat stress | SRR12073757 | SRR12073758 | SRR12073746 | SRR12073735 |
|  | Control | SRR12073729 | SRR12073730 | SRR12073731 | SRR12073732 |
| [SRP268422-B](https://trace.ncbi.nlm.nih.gov/Traces/sra?study=SRP268422) | Heat stress | SRR12073747 | SRR12073748 | SRR12073749 | SRR12073750 |
|  | Control | SRR12073742 | SRR12073743 | SRR12073744 | SRR12073745 |
| [ERP014602-A](https://trace.ncbi.nlm.nih.gov/Traces/sra?study=ERP014602) | Heat stress | ERR1328529 | ERR1328530 | ERR1328531 | ERR1328532 |
|  | Control | ERR1328525 | ERR1328526 | ERR1328527 | ERR1328528 |
| [ERP014602-B](https://trace.ncbi.nlm.nih.gov/Traces/sra?study=ERP014602) | Heat stress | ERR1328545 | ERR1328546 | ERR1328547 | ERR1328548 |
|  | Control | ERR1328541 | ERR1328542 | ERR1328543 | ERR1328544 |
