# Supplementary material for: Gene expression networks and functionally enriched pathways involved in the response of domestic chicken to acute heat stress
Source: Front Genet. 2023 May 2;14:1102136. doi: 10.3389/fgene.2023.1102136 (PMC10185895; doi:10.3389/fgene.2023.1102136)
Supplement: Supplementary file 12 [file Table2.docx]

| Supplementary Table 2. The accession numbers of the used runs for the validation analysis | | | | | | | | | |
| --- | --- | --- | --- | --- | --- | --- | --- | --- | --- |
| Dataset accession number | Group | Accession number of runs | | | | | | | |
| SRP100368 | Heat stress | SRR5273281 | SRR5273282 | SRR5273283 | SRR5273284 | SRR5273285 | SRR5273286 | SRR5273287 | SRR5273288 |
|  | Control | SRR5273273 | SRR5273274 | SRR5273275 | SRR5273276 | SRR5273277 | SRR5273278 | SRR5273279 | SRR5273280 |
| SRP268422-C | Heat stress | ERR1328537 | ERR1328538 | ERR1328539 | ERR1328540 |  |  |  |  |
|  | Control | ERR1328533 | ERR1328534 | ERR1328535 | ERR1328536 |  |  |  |  |
