# Supplementary material for: Gene expression networks and functionally enriched pathways involved in the response of domestic chicken to acute heat stress
Source: Front Genet. 2023 May 2;14:1102136. doi: 10.3389/fgene.2023.1102136 (PMC10185895; doi:10.3389/fgene.2023.1102136)
Supplement: Supplementary file 13 [file Table3.docx]

| Supplementary Table 3. The seventeen most important meta-gens based on seven feature weighting algorithms that obtained a higher average weight when separating samples between acute heat stress and control samples |
| --- |

| Attribute | Weight_Info Gain | Weight_Info Gain Ratio | Weight_Rule | Weight_Chi Squared | Weight_Gini Index | Weight_Uncertainty | Weight_Relief | Average _weight |
| --- | --- | --- | --- | --- | --- | --- | --- | --- |
| TXNDC11 | 1 | 1 | 1 | 1 | 1 | 1 | 0.1 | 0.8663 |
| DERL3 | 1 | 1 | 1 | 0.9 | 1 | 0.9 | 0.1 | 0.8406 |
| SEC23B | 1 | 1 | 1 | 0.7 | 1 | 0.8 | 0.2 | 0.8097 |
| HSPA5 | 1 | 1 | 0.5 | 0.6 | 1 | 0.6 | 1 | 0.809 |
| EIF2A | 1 | 1 | 1 | 0.7 | 1 | 0.8 | 0.1 | 0.7941 |
| ARCN1 | 1 | 1 | 1 | 0.6 | 1 | 0.6 | 0.3 | 0.7893 |
| PRRC1 | 1 | 1 | 1 | 0.7 | 1 | 0.7 | 0.1 | 0.7866 |
| PTPRF | 0.8 | 0.9 | 1 | 0.9 | 0.8 | 0.9 | 0.1 | 0.7824 |
| EIF5B | 1 | 1 | 1 | 0.6 | 1 | 0.7 | 0.1 | 0.7643 |
| SDF2L1 | 1 | 1 | 0.8 | 0.7 | 1 | 0.7 | 0.1 | 0.7586 |
| GUSB | 0.8 | 0.9 | 1 | 0.7 | 0.8 | 0.7 | 0.2 | 0.7526 |
| PPM1K | 0.8 | 0.9 | 0.9 | 0.6 | 1 | 0.6 | 0.4 | 0.7488 |
| UAP1 | 0.8 | 0.9 | 1 | 0.7 | 0.8 | 0.7 | 0.1 | 0.7274 |
| SELENOK | 0.8 | 0.9 | 0.4 | 1 | 0.8 | 0.9 | 0.1 | 0.7273 |
| C6orf62 | 0.8 | 0.9 | 0.4 | 0.8 | 0.8 | 0.9 | 0.3 | 0.7243 |
| USP14 | 0.8 | 0.9 | 1 | 0.6 | 0.8 | 0.6 | 0.2 | 0.7203 |
| PSMC2 | 1 | 1 | 0.7 | 0.4 | 1 | 0.6 | 0.4 | 0.7174 |
